# Blade pitching in vertical axis wind turbines: A double multiple stream tube theoretical approach to performance enhancement

**DOI:** 10.1016/j.heliyon.2025.e42101

**Published:** 2025-01-20

**Authors:** Antim Gupta, Ussama Ali, Hamid Ait Abderrahmane, Isam Janajreh

**Affiliations:** a School of Engineering, Computing, and Mathematics, Oxford Brooke's University, Oxford, United Kingdom; b Department of Mechanical and Nuclear Engineering, Khalifa University of Science and Technology, Abu Dhabi, United Arab Emirates

**Keywords:** Variable blade pitching, Wind turbine, Sustainable energy, Wind energy, Double multiple stream tube (DMST)

## Abstract

This research delves into the performance enhancement of Vertical Axis Wind Turbines (VAWTs) through the innovative approach of variable blade pitching based on Double Multiple Stream Tube theory principles. VAWTs, known for their potential in urban and low-wind environment, often face efficiency and energy yield challenges. This study addresses these challenges by proposing a novel variable blade pitching mechanism that dynamically adapts to changing wind conditions, optimizing the aerodynamic performance, and enhancing the torque and overall performance of VAWT rotor. The efficiency of two pitching models is investigated on 3-bladed NACA0015 rotors, where the blade's local angle of attack is cyclically adjusted below the stall angle to maximize the lift force and torque throughout full revolution. In Model 1, the angle of attack experiences cyclic variation as a sinusoidal function providing smooth pitching, whereas in Model 2, the peak value of angle of attack was fixed below the stall condition forming linear function. The investigation showed substantial improvement in the VAWT performance using both pitching models. The variable blade pitching strategy significantly enhances the lift-to-drag ratio and thus improving the torque output across diverse wind scenarios/tip speed ratios, demonstrating its effectiveness in maximizing the operational efficiency of VAWTs. Blade pitching model 1 and 2 were found to be effective across all lower Tip Speed Ratio (TSR) values, suggesting its robustness in variable wind conditions. A peak average coefficient of performance (Cp) of 0.568 was achieved at TSR = 5 using pitching Model 1 with a maximum angle of attack of 8.5°, compared to a Cp of 0.48 for the fixed blade configuration, near to Betz's limit (Cp = 0.593). The findings confirm that integrating variable blade pitching would substantially improve VAWT performance and offer a clear direction for revolutionary future wind turbine aerodynamic design enhancements.

## Highlights

• Darrieus VAWT performance is assessed under fixed and variable pitching.
• DMST theory coupled with variable blade pitching boosts VAWTs performance.
• TSR signify Tip Speed Ratio which is a common termonology in the Wind Energy Community.
• Variable pitching can be applied in high fidelity CFD model to reveal additional physics.
• Enhanced VAWT performance can contribute to sustainable urban energy solutions.

**Table:** Nomenclature

*Acronym*	*Definition*
BEM	Blade Element Theory
CFD	Computational Fluid Dynamics
DMST	Double Multiple Stream Tube
HWAT	Horizontal Axis Wind Turbine
MST	Multiple Stream Tube
NACA	National Advisory Committee for Aeronautics
SST	Single Stream Tube
VAWT	Vertical Axis Wind Turbine
*Symbol*	*Definition*
*a*	Axial Induction Factor (−)
a_u_	Upstream Induction Factor (−)
a_d_	Downstream Induction Factor (−)
A	Rotor Swept Area (m^2^)
*c*	Blade Chord Length (m)
*C*_*d*_	Coefficient of Drag (−)
*C*_*l*_	Coefficient of Lift (−)
*C*_*m*_	Coefficient of Moment (−)
*C*_*n*_	Normal Force Coefficient (−)
C_p_	Coefficient of Performance (−)
*C*_*t*_	Tangential Force Coefficient (−)
*F*_*t*_	Tangential Force Component (N)
*F*_*n*_	Normal Force Component (N)
*F*_*Tavg*_	Average Tangential Force Component (N)
*F*_*up*_	Upwind Function (−)
*N*	Number of Blades (−)
*N*_*st*_	Number of Stream Tubes (−)
*L*	Turbine Span Length (m)
*P*	Power (W)
*R*	Rotor radius (m)
*S*	Maximum local angle of attack (degree)
*T*	Torque (N/m)
$V_{a_{d}\;}$	Downstream Axial Induction Velocity (m/s)
$V_{a_{u}\;}$	Upstream Axial Induction Velocity (m/s)
$V_{e\;}$	Equilibrium Induced Velocity (m/s)
V_c_	Blade Cordial Velocity (m/s)
V_n_	Blade Normal Velocity (m/s)
V_a_	Induced Axial Velocity (m/s)
$V_{R}$	Local Blade Relative Velocity (m/s)
$V_{R}^{u}$	Local Blade Relative Velocity Upstream (m/s)
$V_{\infty }$	Wind Freestream Velocity (m/s)
*Greek letter*	*Definition*
α	Angle of Attack (degree)
$\alpha^{u}$	Upstream Angle of Attack (degree)
$\alpha^{d}$	Downstream Angle of Attack (degree)
β	Initial Blade Pitch Angle (degree)
λ	Tip Speed Ratio (−)
ρ	Air Density (kg/m^3^)
θ	Blade Azimuth Position (degree)
ω	Rotor Rotational Speed (rad/sec or rpm)
σ	Solidity of Turbine (−)
*ν*	kinematic viscosity (m^2^/s)

## 1 Introduction

The role of wind energy in mitigating global warming is extensively examined in literature [1]. This renewable energy source is being increasingly adopted worldwide due to its cost effectiveness and its compatibility with existing energy systems. Wind energy is a locally available resource that reduces reliance on fossil fuels and lowers CO_2_ emissions. Furthermore, the expansion of both onshore and offshore wind energy sectors support the growth of the green economy [2]. This aligns with the objectives of the COP-28 UAE, and G-7 India Summit, where wind energy was highlighted as a key strategy to reduce greenhouse gas emissions and achieve global sustainable development goals fighting against global climate change [[3], [4], [5]]. To meet these sustainability goals, countries such as the UK and Sweden are working to expand floating offshore energy production due to higher wind intensity near sea level. SeaTwirl, based in Sweden, is currently deploying the Vertical Axis Wind Turbines (VAWTs) in cluster configuration to support government efforts in meeting energy demand goals. VAWTs offer advantages over Horizontal Axis Wind Turbines (HAWTs), particularly due to their faster wake recovery [6].

Over the past few decades, wind turbine technology has advanced significantly, leading to improvement in performance, efficiency, and cost effectiveness. This progress is reflected in the significant increase in global wind energy capacity, which has grown by a factor of 93 over the past two decades [7,8]. The countries leading this surge are China, the USA, and India, collectively accounting for 60 % of the world's wind power generation [9]. In the realm of wind turbine technology, there are two primary types: VAWTs and HAWTs. VAWTs operate based on the aerodynamic principle of airfoil, further categorized into lift-type (Darrieus) and drag-type (Savonius) models. Among these, the Darrieus type VAWT is often regarded as highly efficient, comparable to HAWTs. It is particularly well-suited for urban areas where wind flow can be impeded by buildings, offering benefits such as reduced noise, cost effectiveness, scalability, reliability, and spatial efficiency [10]. In addition, the deployment of VAWTs for tourism purposes on islands could enhance sustainable energy solutions and support eco-friendly tourism initiatives [11].

Investigating the performance and aerodynamic characteristics of Darrieus VAWTs presents a significant challenge due to the complex three-dimensional fluid dynamics involved in blade rotation. This complexity is further intensified by various flow phenomena, including dynamic stall, flow separation, and a lack of self-starting capabilities [12]. To address the complexities of Darrieus VAWTs, several researchers have developed analytical models to assess their steady-state aerodynamic performance. Betz proposed one of the fundamental approaches for turbines, employing classical linear momentum theory to establish the upper limit of the turbine's power coefficient, known as Betz limit, which is a coefficient of performance (Cp) of 59.3 % [13]. Templin further advanced this field by developing the Single Stream Tube (SST) model based on the Actuator Disc theory [14]. However, this model tends to overestimate the performance of Darrieus VAWTs due to simplifying assumptions, such as neglecting aerodynamic stall and airfoil curvature [15].

To improve the accuracy, Strickland refined the actuator disc concept into a model comprising multiple adjacent stream tubes (MST), allowing for the prediction of each stream tube's instantaneous aerodynamic force and induced velocity [16]. Further advancements were made by Paraschivoiu [17] who improved upon the MST model by bifurcating the actuator disc of the rotor into downstream and upstream sections, thus enabling a more precise estimation of axial induction. Additionally, the double-multiple stream tube (DMST) model employed by Paraschivoiu et al. [18], utilized blade element momentum theory (BEM), allowed for the more accurate prediction of axial induction velocity at various azimuth positions of the blade within the stream tube. Concurrently, Vandenberghe et al. [19], and Hirsch et al. [20] proposed theoretical models based on vortex and cascade theory, respectively, to predict the wind turbine's steady-state performance. Although these models are computationally intensive, they remain more cost-effective than high-fidelity Computational Fluid Dynamics (CFD) methods and experimental approaches such as wind tunnel or field testing [21,22].

The “dead band” phenomenon presents a notable challenge in the operation of VAWTs, particularly pertinent at low wind velocities. This issue is characterized by the inability of the turbine to self-start due to a negative value or almost zero value for Cp, occurring primarily at wind speeds (v) below 5 m/s. This limitation significantly impedes the turbine's capacity to extract power efficiently in regions with consistently low wind velocities. Furthermore, The Cp is found to be lower at low wind speed conditions. A key factor exacerbating this issue in VAWTs is the boundary layer separation and vortex shedding occurring at the airfoil blade surface. This problem is particularly pronounced when the blades are subjected to a high angle of attack (AoA) [23]. Moreover, these turbine's fixed blades design configuration contributes to their inability to self-start, especially at low tip speed ratios (ranging from 1 to 3). Given these challenges, enhancing the overall performance of Straight-Bladed Vertical Axis Wind Turbines (S-VAWT) is essential. A viable approach to addressing this issue is adjusting the blade pitch angle. Adjusting the blade pitch angle offers a promising solution, as optimizing this angle can reduce fluid flow separation effects, thereby enhancing turbine efficiency and improving self-starting capabilities at low wind speeds. A study conducted by Kanyako and Janajreh [24] based on DMST method validated with Paraschivoiu's work [25], demonstrated that VAWTs operating at low TSRs (λ = 1–3) suffer from negative or near to zero power coefficients (Cp) due to reduced aerodynamic effectiveness.

Extensive research has focused on enhancing the efficiency of H-rotor VAWTs by identifying critical design factors that influence performance. Studies have shown that variable blade pitching, in particular, can significantly improve turbine efficiency, self-starting capability, and overall aerodynamic stability, as confirmed by numerous experimental and computational analyses [[26], [27], [28]]. Dumitrache et al. [26] explored initial pitching of straight-bladed VAWTs rotor and found that implementing variable pitch can dramatically enhance self-starting abilities and reduce the time required for turbines to reach optimal rotational speed. Despite such findings, much of the literature still concentrates on fixed or initial blade pitching configurations. Recent studies continue to underscore that controlled modification of blade pitch angles can lead to marked improvements in performance [[29], [30], [31], [32]].

For example, Sagharichi et al. [29] applied pitch control strategies specifically within low Tip Speed Ratio (TSR) ranges, demonstrating that a negative pitch angle of -π/6 could reduce flow separation, lower torque ripple, and thus improve overall turbine efficiency. Liang et al. [30] also highlighted the role of blade pitching in enhancing self-starting capabilities, reporting a 20 % increase in performance at a TSR of 1.5 through adjustments aimed at maximizing lift at each rotor azimuth angle. Similarly, Xu et al. [31] studied influence of fixing initial blade angle and noticed a 78 % performance enhancement using optimal pitch function using wind tunnel. Sun et al. [32] took this further by fine-tuning both the offset pitch angle and blade position, which minimized vortex separation and improved the turbine's power output.

Passive blade pitching using CFD has also gained attention for its ability to enhance efficiency through minimal design changes. Hammad et al. [33] used Simulink to demonstrate that initial blade pitch adjustments could boost self-starting ability by up to 80 % with pitch regulation. Peng et al. [34] employed passive toe-out pitching in a wind tunnel, achieving peak performance at a −6.5° angle. In addition, Xu et al. [35] studied fixed pitch angles on a NACA 2418 rotor, revealing that 10° and 5° pitch angles reduced self-starting times by 20 % and 12 %, respectively. Xu et al. [36] examined dynamic pitching control, finding that variable pitching for paired rotor improved total power output by 20 %. Furthermore, Shaheen [37] studied variable blade pitching for paired rotors, achieving a 26 % improvement for a two turbine cluster over standalone turbines and a 38 % increase in Cp for three turbine cluster using slight modification in local angle of attack. Zhang et al. [38] used CFD to investigate fluid-solid interaction and integration of variable blade pitching for TSR values of 0.1 and 3.5, recording a 16.42 % performance increase. Su et al. [39] analyzed 3D CFD simulations of VAWTs under pitching motion, reporting an increase in Cp of 1.5 %–15 % alongside enhanced aerodynamic stability.

Optimization-based approaches have also shown promise as a way to enhance VAWTs performance. Zhang et al. [40] reported a 15 % improvement through blade pitching adjustments; however, their study focused on a single-blade design at specific TSR values, utilizing computationally intensive optimization methods. Similarly, Abdalrahman et al. [41] combined artificial neural networks (ANN) with CFD simulations to model blade pitching, achieving a 22 % performance increase, though the approach was computationally demanding.

Recent advancements in wind energy technology have sparked a heightened interest in applying variable pitching techniques for wind turbines. A notable approach within this domain involves the strategic modification of the turbine blade's local angle of attack, both upstream and downstream, as a means to optimize performance [42,43]. In the context of VAWTs, the significance of blade pitching in performance enhancement, particularly under low wind conditions, cannot be overstated. To this end, conducting sensitivity analyses focused on power augmentation in such conditions is crucial. Here, the primary objective is to ascertain the optimal local AoA for the rotor blades that will maximize the tangential force and thus produce more lift force on blade, thereby substantially improving the performance of Darrieus turbines. Variable blade pitching strategies are employed in H-rotor VAWTs to achieve this objective. A fast-numerical approach was developed using the DMST theory to predict the performance of VAWTs. The developed model was validated and coupled with two blade pitching models targeting an improvement in wind turbine efficiency. The findings from this research not only strengthen existing studies on wind turbines but also have significant potential to transform the wind turbine industry, enhancing rotor performance in clustered configurations for both offshore and onshore applications.

## 2 Methodology

**Fig. 1:**
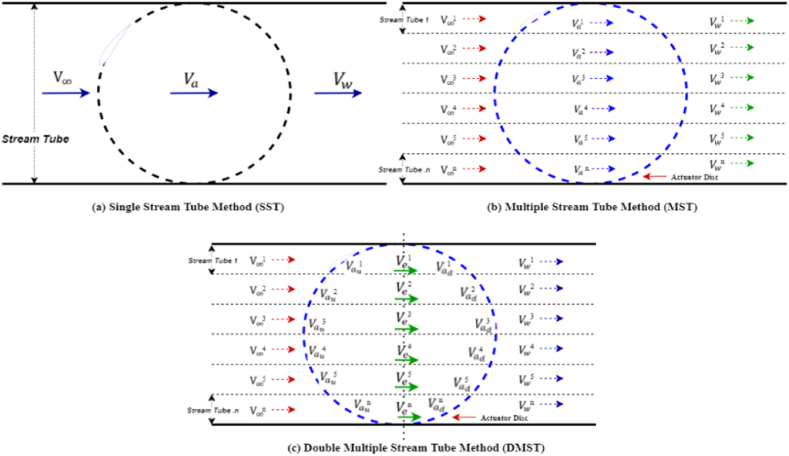
a) Single Stream Tube, b) Multiple Stream Tube, and c) Double Multiple Stream Tube models.

The Darrieus VAWT's rotor comprises of aerodynamically designed turbine blades to extract kinetic energy from wind based on lift principles. To investigate the intricate aerodynamic characteristics and performance of Darrieus VAWT, various models have been developed, including Multiple Stream Tube (MST), Double Multiple Stream Tube (DMST), cascade, vortex, and panel methods [44]. Each model offers its own pros and cons that vary based on the wind conditions, wind turbine configuration, and the computational runtime. VAWTs prediction models can be categorized into three main types: Momentum, Vortex, and CFD [28]. The blade element momentum (BEM) model is used in this study to predict the turbine's performance. This method combines classical Blade Element Theory (BET) with momentum actuator disc theory together to form Double Multiple Stream Tube theory. Fig. 1 (a, b, and c**)** illustrate single-, multiple-, and double multiple stream tube methods, respectively.

### 2.1 Aerodynamics of straight bladed VAWT

**Fig. 2:**
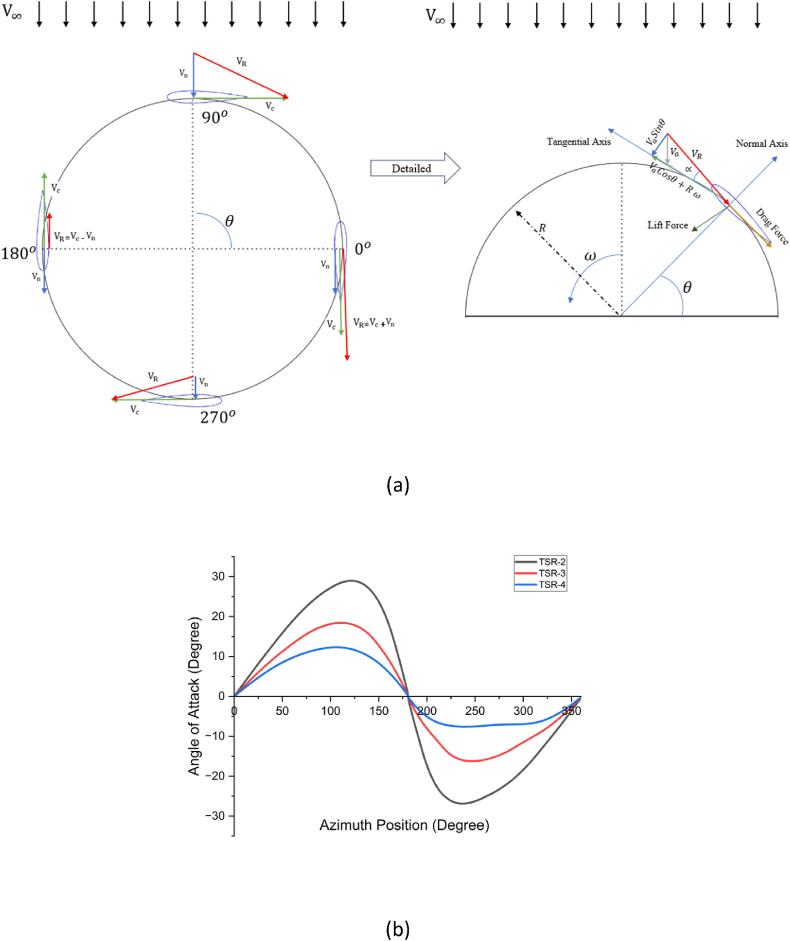
a) Velocity and force diagram for Darrieus VAWT rotor, b) Variation of local angle of attack (AoA) with respect to azimuth position at tip speed ratios (TSR) of 2, 3 and 4.

The straight blade of Darrieus VAWT rotor presents a challenge due to its complex aerodynamic behavior. The complexity results from the fact that downstream- and upstream-induced velocities are not known. The airfoil blade serves as a barrier, absorbing or exchanging a portion of the wind energy to generate torque, which in turn reduces the wind velocity intensity in upstream, mid-stream and downstream regions of rotor. This decrease in axial velocity is referred to as the axial induction velocity, and the decrement factor is known as the induction factor [17]. As depicted in Fig. 2, the relative velocity ($V_{R})$ is calculated using normal $\left(V_{n}\right)$ and cordial velocity $\left(V_{c}\right)$ components, calculated using Eqs. (1)–(3):

(1) $$V_{c}=V_{a}\;\cos\;\theta +R\omega$$

(2) $$V_{n}=V_{a}\;\sin\;\theta$$

(3a) $$V_{R}=\sqrt{V_{c}^{2}+V_{n}^{2}}$$

(3b) $$V_{R}=\sqrt{\left({\left(V_{a}\;\sin\;\theta \right)}^{2}+{\left(V_{a}\;\cos\;\theta +\omega R\right)}^{2}\right)})$$

where *R* is the radius of turbine rotor (m), ω is the rotor's rotational velocity (rad/sec), θ is the blade azimuth angle (degree), $V_{a}$ is the induced axial velocity (m/s). By normalizing the relative velocity with freestream velocity $\left(V_{\infty }\right)$ and utilizing the definitions of the tip speed ratio ($TSR\;or\;\lambda =R.\omega /\;V_{\infty })$ and axial induction factor ($a=V_{a}/V_{\infty })$ leads to non-dimensional Eq. (4)**:**

(4) $$\frac{V_{R}}{V_{\infty }}=\sqrt{{\left(\left(1-a\right)\sin\;\theta \;\right)}^{2}+{\left(\left(1-a\right)\cos\;\theta +\lambda \right)}^{2}}$$

Based on Fig. 2, the angle of attack−*α* (AoA) and its change over time can be determined by examining the blade's azimuth position and tip speed ratios (λ or TSR), as outlined in Eq. (6). It is important to note that this equation was derived through manipulations of Eqs. (3)–(5).

(5) $$\mathrm{Tan}\;\alpha =\frac{V_{a}\;\sin\;\theta }{V_{a}\;\cos\;\theta +\omega R}$$

(6) $$\alpha =Tan^{-1}\left(\frac{\left(1-a\right)\sin\;\theta }{\left(1-a\right)\cos\;\theta +\lambda }\right)$$

The evolution of *α* is depicted in Fig. 2 (b) for different TSRs (2, 3, and 4). It shows that the local AoA decreases with increased TSR for all rotor azimuth angles. It is used to estimate local variation in tangential ($C_{t}$) and normal ($C_{n}$) force coefficients using Eqs. (7), (8), respectively:

(7) $$C_{t}=C_{l}\;\sin\;\alpha -C_{d}\;\cos\;\alpha$$

(8) $$C_{n}=C_{l}\;\cos\;\alpha +C_{d}\;\sin\;\alpha$$

where the coefficients of lift ($C_{l})$ and drag ($C_{d})$ in Eqs. (7), (8) are interpolated based on large aerodynamic data set fed into DMST model containing Reynolds number (Re_*L*_) versus *α*_*L*_. The tangential and normal force components of each blade at any instantaneous azimuth position are calculated using Eqs. (9), (10):

(9) $$F_{t}=\frac{1}{2}\rho V_{R}^{2}\left(hc\right)C_{t}$$

(10) $$F_{n}=\frac{1}{2}\rho V_{R}^{2}\left(hc\right)C_{n}$$

where, *ρ* represents the density of air density ($kg/m^{3}$), *c* is the chord length of the airfoil (m), and ℎ is the vertical height of the airfoil (m). The normal and tangential force components vary with the rotor azimuth angle (*θ*). The tangential force component primarily contributes to torque in the turbine, and the average tangential force for a single airfoil is calculated using Eq. (11):

(11) $$F_{T_{avg}}=\frac{1}{2\pi }\int_{0}^{2\pi }F_{t}\left(\theta \right)\;d\theta$$

The total torque generated by the wind turbine can be expressed using Eq. (12):

(12) $$T=NRF_{T_{avg}}=\frac{NR}{2\pi }\int_{0}^{2\pi }F_{t}\left(\theta \right)\;d\theta$$

where *N* is the number of airfoils. The torque value determines the total turbine output power, which is normalized as the power coefficient $C_{p}$, given as Eqs. (13), (14), respectively. The $C_{p}$ is defined as the ratio of the rotor's absorbed power to the total power available in the incoming wind stream.

(13) $$P_{output}=T\omega =\frac{\omega NR}{2\pi }\int_{0}^{2\pi }F_{t}\left(\theta \right)\;d\theta$$

(14) $$C_{p}=\frac{P_{output}}{P_{available}}=\frac{T\omega }{\frac{1}{2}\rho AV_{\infty }^{3}}.\frac{\omega R}{V_{\infty }}=C_{m}.\lambda$$

Where *A* ${\text{and}\;C}_{m}$ are the projected area of the turbine (m^2^), and the moment coefficient, respectively.

### 2.2 Double multiple stream tube

This method was introduced by Paraschivoiu et al. [17] to estimate wind turbine performance. In this method, forces on turbine blades are assessed using momentum theory, which applies the basic principles of conservation of angular and linear momentum within a control volume. Conversely, Blade Element Momentum (BEM) theory analyzes forces on a small blade segment. This combined method is also known as the strip theory [45]. The DMST model integrates the MST model with double actuator disk theory to predict turbine performance.

**Fig. 3:**
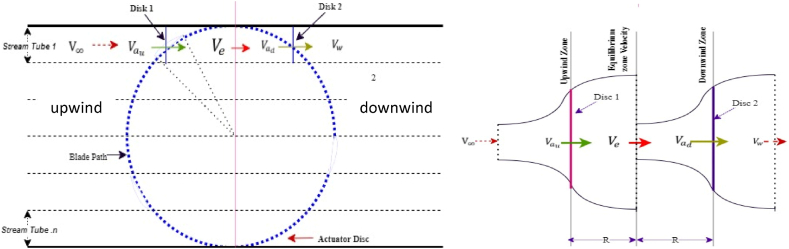
Schematic of double multiple stream tube method.

Referring to Fig. 1, the rotor actuator disc in the DMST model is split into two zones: the upstream half and the downstream half as shown in Fig. 3. Each zone is further divided into multiple parallel stream tubes. The aerodynamically designed blade absorbs kinetic energy of wind and converts it into useful mechanical torque, reducing the freestream velocity by axial induction factors *a*_*d*_ and *a*_*u*_ for the downstream and upstream regions, respectively, along the stream tube's axial direction. Consequently, the downstream-induced ($V_{ad}$), upstream-induced ($V_{au}$), and equilibrium-induced ($V_{e}$) velocities are also reduced, respectively expressed in Eqs. (15), (16), (17) as:

(15) $$V_{a_{d}}=a_{d}\left(2a_{u}-1\right)V_{\infty }$$

(16) $$V_{a_{u}\;}=a_{u}{\;V}_{\infty }$$

(17) $$V_{e}=\left(2a_{u}-1\right)V_{\infty }$$

The calculation is carried out separately for each stream tube in both the downstream and upstream half-cycle zones. Additionally, BEM theory is applied to derive the aerodynamic forces on the blades, facilitating the estimation of total output power and mechanical torque. Lastly, using fundamental blade element theory, local angle of attack and relative wind velocity are calculated, as shown below:

For the upstream region (π/2 < θ < -π/2):

(18) $$V_{R}^{u}=\sqrt{\left({\left(V_{a_{u}}\;\sin\;\theta \right)}^{2}+{\left(V_{a_{u}}\;\cos\;\theta +R\omega \right)}^{2}\right)})$$

(19) $$\alpha^{u}=Tan^{-1}\left(\frac{\left(1-a_{u}\right)\sin\;\theta }{\left(1-a_{u}\right)\cos\;\theta +\lambda }\right)$$

For the downstream region (-π/2 < θ < π/2):

(20) $$V_{R}^{d}=\sqrt{\left({\left(V_{a_{d}}\;\sin\;\theta \right)}^{2}+{\left(V_{a_{d}}\;\cos\;\theta +R\omega \right)}^{2}\right)})$$

(21) $$\alpha^{d}=Tan^{-1}\left(\frac{\left(1-a_{d}\right)\sin\;\theta }{\left(1-a_{d}\right)\cos\;\theta +\lambda }\right)$$

Using the local angle of attack and relative velocity, the updated induction factor for each stream tube is calculated by equating the forces derived from the momentum equation with those obtained from blade element theory:

(22) $$F_{up}\left(1-a\right)=\pi a$$

where the upwind function $F_{up}$ is given as:

(23) $$F_{up}=\frac{Nc}{8\pi R}\int_{-\frac{\pi }{2}}^{\frac{\pi }{2}}{\left(\frac{V_{r}}{V_{a}}\right)}^{2}\left(C_{n}\frac{\cos\;\theta }{\left|\cos\;\theta \right|}-C_{t}\frac{\sin\;\theta }{\left|\sin\;\theta \right|}\right)\;d\theta$$

This method is executed using an iterative solver. Once the correct relative velocity, induction factor, and AoA are determined, the data can be utilized to predict the C_p_ (as shown in Eq. (24)), along with other coefficients. By evaluating the C_p_ for both downstream and upstream zones, the overall C_p_ of the turbine can be determined.

(24) $$C_{p}=\frac{Nch}{2\pi R}\int_{-\frac{\pi }{2}}^{\frac{\pi }{2}}{\left(\frac{V_{r}}{V_{a}}\right)}^{2}C_{t}\;d\theta$$

**Fig. 4:**
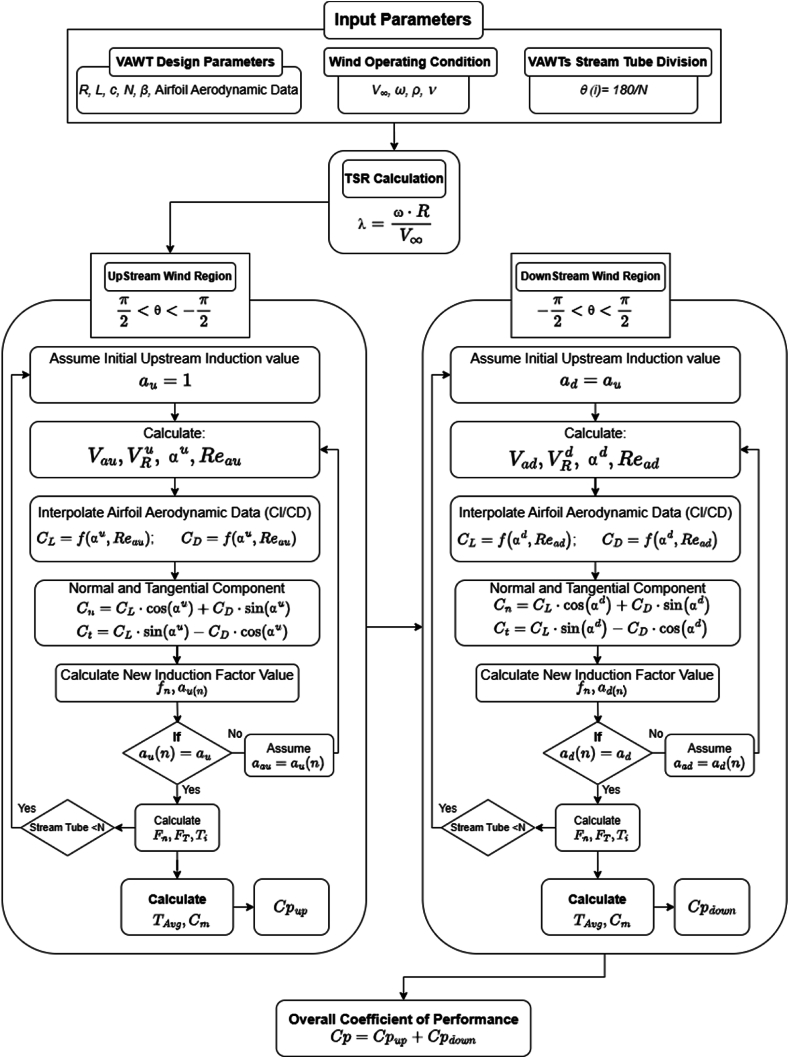
Double Multiple Stream Tube algorithm.

The implementation of a DMST algorithm based on the strip theory (BEM) is discussed in this work. A MATLAB script based model was developed and validated against the work of Paraschivoiu et al. [25]. Fig. 4 illustrates the procedure underlying the proposed DMST model. The algorithm segments the upstream and downstream zones of the stream tube into *N* spatial divisions, each containing multiple horizontal parallel stream tubes. Input parameters such as freestream velocity (*V*_*∞*_), blade pitch angle (*β*), spatial divisions (*Nh=*180*/N*), turbine's rotor speed (*ω*), and airfoil data ($Re,C_{d},C_{l})$ are considered. The aerodynamics data for each airfoil subjected to various Reynolds numbers (Re_*L*_) ranging from 2 × 10^4^ to 10 × 10^7^ is feeding into the program, and this aerodynamics data is extracted from XFLR5 (airfoil analysis software). The algorithm calculates the C_p_ for each stream tube. An iterative solver is used accurately to determine the axial induction factor values (*a*_*u*_ and *a*_*d*_) for each stream tube, with a tolerance value of 1 × 10^−6^.

It should be noted that the algorithm does not consider variations in the viscous properties of the fluid, vertical height of the turbine, flow separation or vortex shedding around the blade. The model is based on basic blade element theory and does not incorporate the governing Navier-Stokes equations for fluid flow. The algorithm employs an iterative approach to estimate the induction factor value for each stream tube in the downstream and upstream zones. By balancing the forces calculated from the momentum equation with those from the blade element theory, the algorithm begins with an initial guess. It then evaluates the blade angle of attack, induced velocity, normalized velocity, and Reynolds number for a given rotor azimuth angle. The algorithm interpolates $C_{l}$ and $C_{d}$ using *α* and Re_*L*_ aero data for each stream tube and estimates the normal force coefficient ($C_{n}$) and tangential force coefficient ($C_{t}$). The moment of coefficient is subsequently determined using actuator blade theory and aerodynamic force, which is then utilized to calculate the updated induction factor value. The iterative solver checks the residual value of the induction factor and minimizes the error or the induction factor imbalance. This procedure is repeated for every stream tube in the downstream and upstream regions. The total performance of the turbine is calculated using the average *C*_*p*_ value for each stream tube, as shown in Fig. 4.

### 2.3 Integration of variable blade pitching model into DMST code

To incorporate variable blade pitching into the DMST, independent pitching models (Model 1 and Model 2) were developed and directly integrated into the validated DMST code. The proposed blade pitching models, developed based on blade element theory, were adapted from our previous research on blade pitching in VAWTs using a CFD approach [46]. In H-rotor VAWTs, each turbine blade undergoes cyclic variations in local angle of attack and blade tip speed, as described by Eqs. 6 and 3, respectively.

**Fig. 5:**
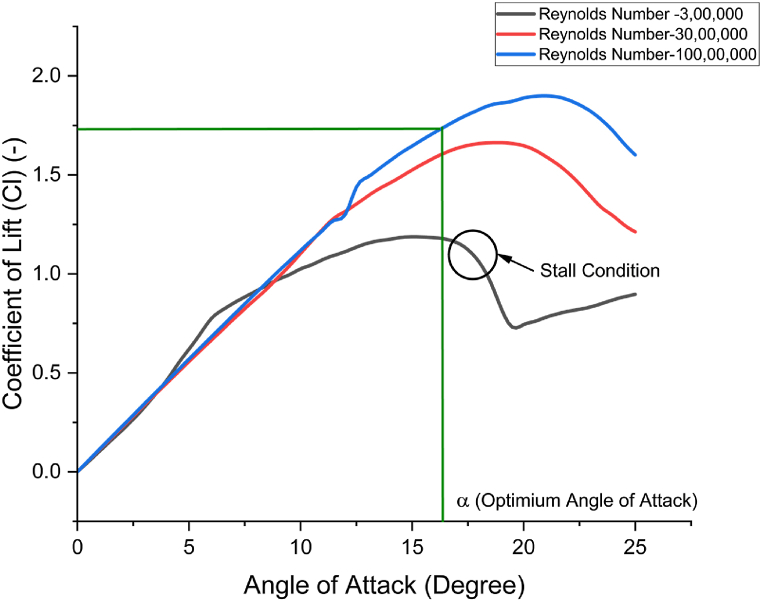
Lift coefficient ($C_{l}$) for NACA0015 airfoil subjected to different Angle of Attack and Reynolds Numbers (*Re*).

Any change in the local angle of attack (*α*_*L*_) influences the overall performance of the turbine. According to aerodynamic principles, these turbines generate torque through tangential force, primarily the lift force experienced by each blade. In lift-based turbines, this lift force is highly dependent on the local angle of attack. The maximum lift coefficient ($C_{l}$) is achieved at a critical value of *α*_*L*_. However, further raising *α*_*L*_ results in a sudden drop in lift and a sharp rise in drag, leading to the stall, as illustrated in Fig. 5.

By setting the blade's peak angle of attack ($\alpha_{L})$ to just below the stall during the full cyclic revolution, VAWT's performance can be improved. An active blade pitching system can be employed to achieve this, enabling the turbine blades to continuously maintain the optimal local pitch angle below the stall in both the downstream and upstream zones. Based on this concept, the optimal blade pitch angle of attack ($\alpha_{L})$ was proposed using the following function:

(25) $$\alpha_{L}=S\times \sin\;\theta$$

where *S* denotes the maximum pitch angle below stall, it is hard-coded within the DMST model as a user-defined function. At lower TSR values, generally the peak *α*_*L*_ tends to be significantly larger than the stall angle, resulting in the blades generating less lift and higher drag, thereby causing a decline in the performance. In such scenarios, the method of variable pitching can be employed to run the turbine at a reduced angle of attack, thus avoiding a stall condition that would otherwise negatively impact the turbine's performance. In this research, two pitching models were developed: Model 1 was based on Eq. (25) as a sinusoidal function of AoA variation, and Model 2 was developed by locally widening the AoA using Model 1 in form of linear function. In Model 1, the angle of attack was varied at every blade-azimuth position, while in Model 2, the blade pitching was adjusted in such a way that the peak angle of attack was kept close to the optimum value at most blade-azimuth positions.

**Fig. 6:**
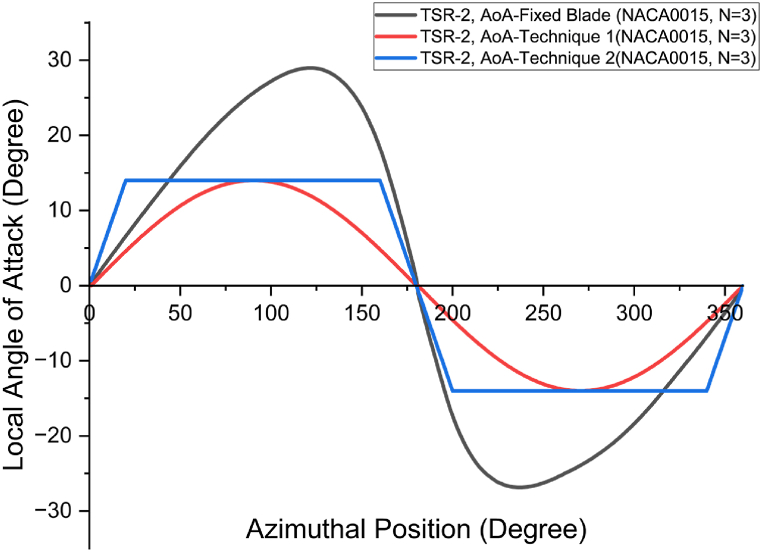
Local angle of attack for fixed blade and variable pitch models 1 and 2 at TSR = 2 as a function of the blade azimuth location.

Fig. 6 illustrates the cyclic variation of the local *α*_*L*_ as a function of blade azimuth position at a Tip Speed Ratio (TSR) of 2. Utilizing the maximum and minimum values of Reynolds number, the optimal AoA was determined that generates the max desired lift coefficient. This optimal AoA is denoted by *S*, represented by the red line in Fig. 6, corresponding to pitching technique 1 (Model 1). The local range of $\alpha_{L}$ was broadened to consistently position the airfoil just before the stall, representing pitching technique 2 (blue line in Fig. 6). The $\alpha_{L}$ was adjusted to zero at blade positions of 0°, 180°, and 360°, corresponding to the smooth transition points for blade in the upward and downward regions.

**Fig. 7:**
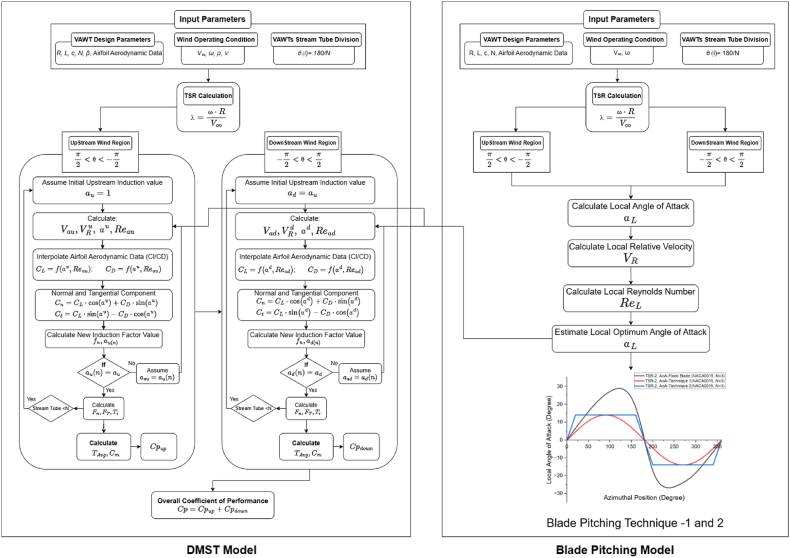
The flow chart for modified DMST algorithm integrating blade pitching technique.

The DMST code was modified and the upstream and downstream AoA (blue line in Fig. 6) was replaced with the optimum AoA (red line for Model 1, and blue line for Model 2), illustrated in Fig. 6. The modified algorithm flowchart for DMST is illustrated in Fig. 7.

## 3 Results and discussion

**Fig. 8:**
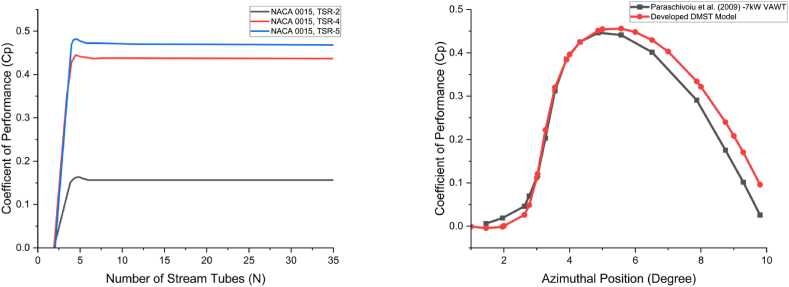
Validation of the developed DMST model: a) Influence of the number of tubes (left), b) Comparison with 7 kW VAWTs Paraschivoiu's model [25] (right).

To assess the predictive accuracy of our DMST model, we initially conducted a stream tube sensitivity test using the turbine design parameters outlined in Table 1. For precise variable blade pitch control, a linear discretization of 180 stream tubes was applied for upstream and downstream zones of the rotor, enabling smooth blade pitching and facilitating an accurate evaluation of the turbine's performance. As illustrated in Fig. 8(a), the Coefficient of Performance (Cp) is shown as a function of the number of stream tubes in the DMST model for three different TSR values (2, 4, and 5). Our results indicate that using more than twenty stream tubes is essential for reliable Cp predictions, even without the integration of blade pitch adjustments. Notably, when the model is limited to only two stream tubes, Cp values approach zero or become negative across all Tip Speed Ratios (TSRs), underscoring the inadequacy of low tube counts for accurate performance representation. Our findings demonstrate that a minimum of twenty stream tubes ensures that Cp values converge consistently to an asymptotic level, achieving reliable performance predictions for the VAWT, even in the absence of variable blade pitching. This establishes a practical threshold for stream tube discretization, ensuring that performance predictions remain robust and independent of the number of stream tubes used in the model.

**Table 1:** Model parameters used for validation [25].

Variable	Symbol	Unit	Value
Airfoil type	–	–	NACA0015
Blade chord length	*c*	m	0.2
Density	*ρ*	kg/m^3^	1.225
Freestream velocity	$V_{\infty }$	m/s	1.95-19.5
Initial angle of attack	*β*	degree	0
Number of blades	*N*	–	2
Pressure	*P*	Pa	101325
Rotor radius	*R*	m	3
Tip speed ratio	TSR or λ	–	1–10
Turbine speed	ω	rpm	125
Vertical height	*h*	m	6

The developed model was then validated against the experimental results of Paraschivoiu et al. [25], conducted on a 7 kW standalone turbine model using the turbine design parameters provided in Table 1. As illustrated in Fig. 8(b), our DMST model closely aligns with Paraschivoiu's [25] results, demonstrating similar trends in Cp across a range of TSRs. This strong correlation confirms the accuracy of our model in predicting VAWT performance under various conditions. While the model performs well across most TSRs, minor discrepancies appear at higher TSRs (TSR >5). These deviations are attributed to slight variations in the interpolated drag ($C_{d}$) and lift coefficient ($C_{l}$), which were adjusted to cover the broader range of Angles of Attack (AoA) used in this study. Such adjustments were necessary to extend the model's applicability.

Our comprehensive study investigates the effectiveness of incorporating variable blade pitching in enhancing the performance of VAWTs using the DMST theory. We examine two distinct blade-pitching techniques (Model 1 and 2) across various wind conditions, focusing on their effects on a NACA0015 3-bladed rotor configuration, with turbine design parameters detailed in Table 1. The data, derived from a series of computational experiments at different TSR, elucidate the dynamic behavior of moment/performance coefficient and angle of attack throughout the rotor's revolution, revealing key findings discussed in the following sections.

### 3.1 Blade pitching technique effectiveness

**Fig. 9:**
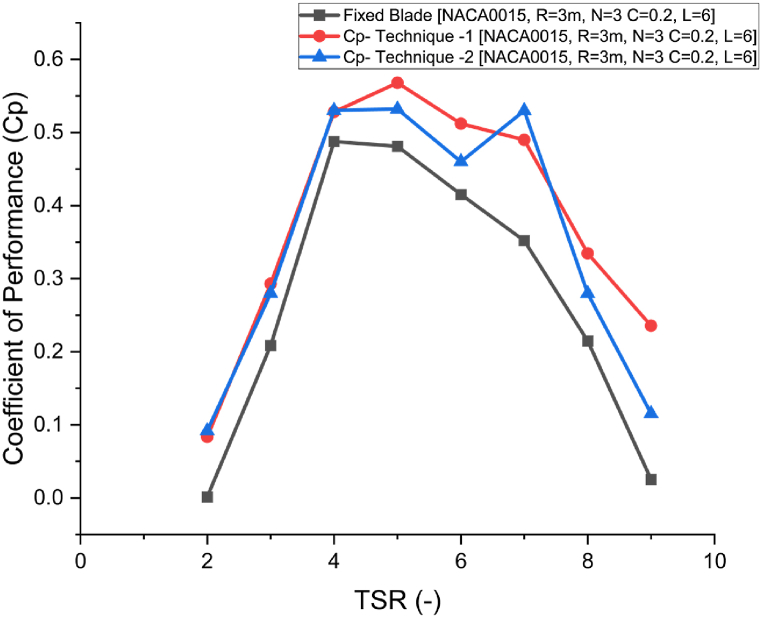
Comparison of Cp against different TSR from 2 to 9 for fixed blades compared against active blade pitching model techniques 1 and 2.

**Fig. 10:**
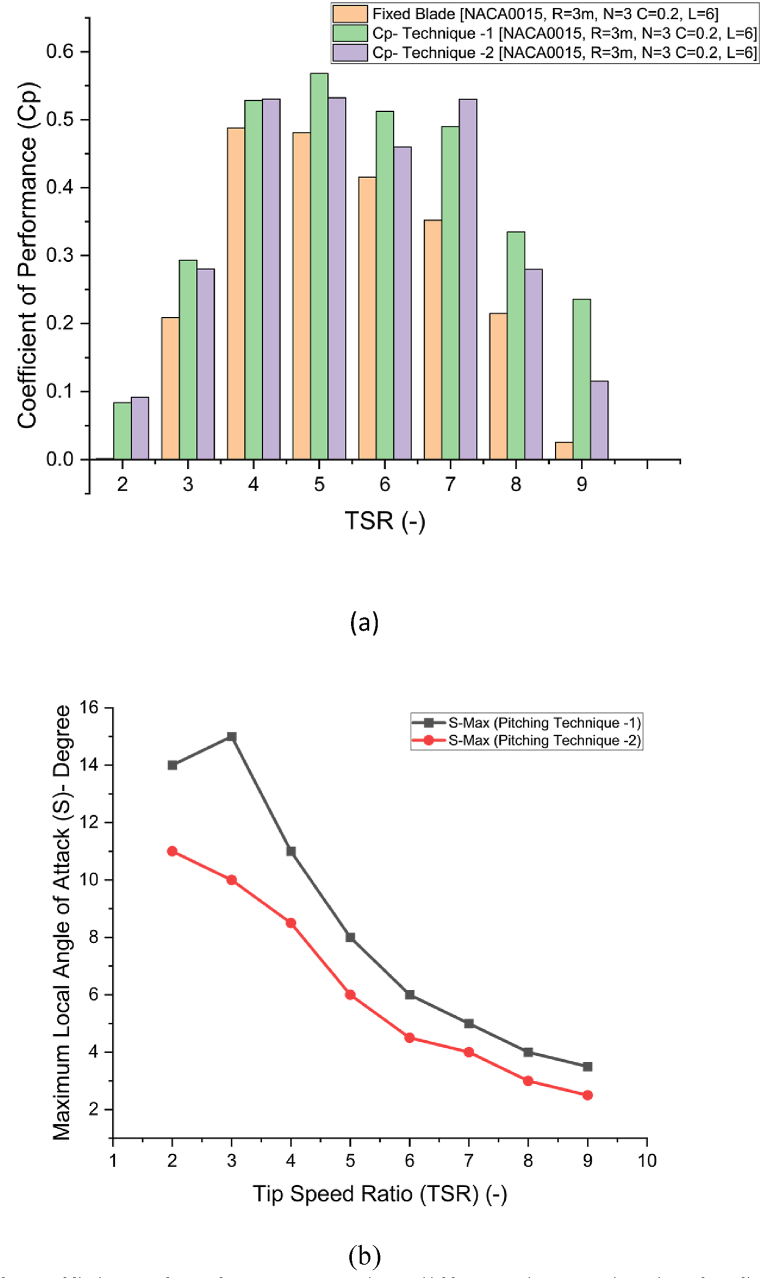
a) Comparison of Coefficient of Performance against different tip speed ratios for fixed blade with pitching model technique 1 and 2, b) Maximum local angle of attack (*S*) used during technique 1 and 2 to achieve Cp in Fig. 10(a).

As shown in Fig. 9, both variable blade pitching techniques (1 and 2) significantly improve the performance (Cp) of the fixed-blade rotor configuration across the TSR range from 1 to 9, as illustrated in Fig. 9, Fig. 10. The results indicate that dynamic blade pitching enhances wind energy capture for a 3-bladed NACA rotor by adjusting the blade's local angle of attack throughout its cyclic revolution, keeping it near an optimal value below the stalling condition of the NACA0015 airfoil, as shown in Fig. 10(b). This adjustment minimizes fluid flow separation and reduces aerodynamic drag, allowing smoother airfoil performance and more efficient energy conversion.

Reducing flow separation is crucial as it prevents the formation of turbulent wake regions, which typically increases drag and reduces lift. By maintaining optimal AoA values and avoiding blade stalling, both techniques keep the blades in an aerodynamic state that maximizes lift throughout each rotation. This lift force translates directly into greater tangential force, contributing to improved torque and higher overall Cp.

Both techniques achieve similar performance enhancement in the TSR range from 2 to 4 due to increased lift generation. However, Technique 1, which uses a sinusoidal modification in local AoA, produces slightly higher Cp values than Technique 2 at TSR values of 5 and 6. A peak Cp of 0.568 (approaching Betz's limit) was achieved at TSR = 5 using Technique 1, compared to peak Cp of 0.48 for the fixed-blade configuration, with an optimal pitch angle *S* of 8.5°. For Technique 2, Cp reached 0.532 at *S* = 6° and TSR = 5. At TSR = 7, Technique 2 achieved peak Cp of 0.53 at *S* = 4°, compared to 0.35 for the fixed-blade VAWT, and 0.49 for Technique 1 at *S* = 5°. This sustained lift generation and minimal flow separation at higher TSRs emphasize Technique 2's effectiveness in high-speed conditions.

At high TSR values of 8 and 9, corresponding to high wind speeds, variable blade pitching remains effective, with both techniques performing well. However, Technique 1 demonstrates slightly better performance than Technique 2. Technique 1 provides smoother blade pitching due to its sinusoidal function, while the linear variation in Technique 2 results in a slight reduction in Cp. The findings suggest that a higher pitch angle is beneficial at lower wind speeds to capture wind energy and maximize lift, maintaining a high Lift/Drag ratio and enhancing blade torque efficiency. At higher TSRs, a slight reduction in maximum *S* boosts performance by minimizing drag and maintaining consistent lift, as shown in Fig. 10(b).

The results also show that at TSR = 2, fixed-blade turbines face issues with dead band or self-starting, resulting in a Cp of zero. However, variable blade pitching improves the self-starting capability of VAWTs, achieving a Cp of approximately 0.1 with both techniques. This improvement occurs because variable pitching helps maintain the necessary lift for rotation initiation, even at low wind speeds, by reducing flow separation and aligning the blade more effectively with the wind direction. These findings underscore the advantage of dynamic blade pitching in enhancing VAWT performance across varying wind speeds and TSR values, particularly by reducing fluid flow separation and maximizing lift. Similar findings have been reported in the literature [46]. It is also noteworthy that the combined effect of each blade's pitching contributes to the overall performance improvement of VAWTs.

### 3.2 Dynamic response

**Fig. 11:**
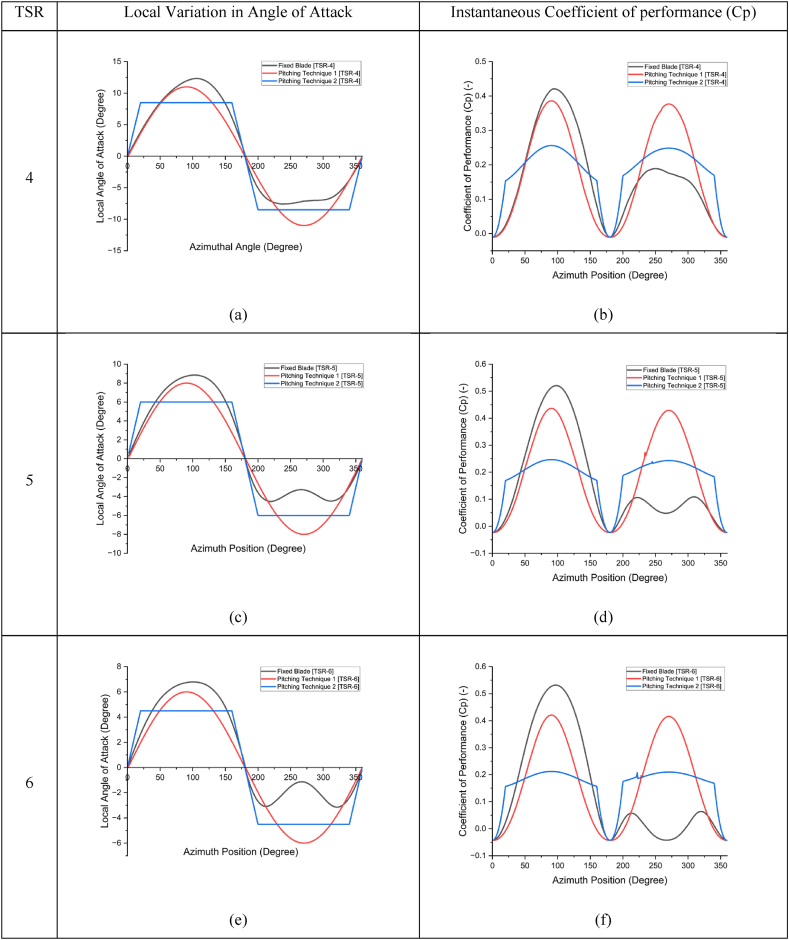
Instantaneous Coefficient of Performance (Cp) as a function of one blade azimuth position obtained using Variable blade pitching technique 1 and 2 compared to fixed 3 blade rotor configuration at TSR 4 (a, b), 5(c, d), and 6 (e, f).

Fig. 11 illustrates the instantaneous Cp (see Fig. 11b–d, and f) of a single blade in a 3-blade rotor configuration at TSR values of 4, 5, and 6 using pitching Techniques 1 and 2 (see Fig. 11a–c, and e), compared to the fixed-blade configuration. It shows that the local angle of attack decreases as TSR increases, with peak upstream AoA values of 13, 9, and 7° at TSR values of 4, 5, and 6, respectively. In the downstream region, reduced peak AoA values of 6, 5, and 4.5° were observed for TSR values of 4, 5, and 6, respectively, due to variations in relative blade velocity. This reduction occurs as the blade absorbs kinetic energy and acts as a blockage, causing a difference in induction factors between the upstream and downstream regions.

The integration of Techniques 1 and 2 allows for variations in local AoA on the fixed-blade configuration to assess model effectiveness. Optimum peak AoA values, or *S*, were fixed at 11, 8, and 6° for Technique 1 and at 8.5, 6, and 4.5° for Technique 2 in the upstream and downstream regions for TSR values of 4, 5, and 6, respectively. Here, induction factor effects on local AoA variation were not considered, and Cp was monitored for a single blade of a 3-bladed NACA0015 rotor.

In the fixed-blade configuration, peak Cp values occur in the upstream region, with lower values in the downstream region due to reduced blade relative velocity. This is shown by the black lines in Fig. 11 (b, **d**, and **f)**. Implementing the pitching model substantially increased the performance coefficient, indicating improved torque in both the upstream and downstream regions of the rotor. This enhancement, combined with the blade count, suggests more efficient energy capture and torque distribution throughout the rotation cycle.

Technique 1, represented by the red line, achieves the highest peak Cp values, particularly in the upstream region. This implies that Technique 1 effectively optimizes AoA to maximize lift and reduce drag, resulting in greater torque. The sinusoidal AoA modification helps align the blades with the wind direction, crucial for efficient energy capture, especially in high-wind upstream areas.

Technique 2, shown by the blue line, produces a smoother Cp curve with less fluctuation between upstream and downstream regions. While its peak Cp is slightly lower than Technique 1, Technique 2 provides more consistent performance across the cycle. This stability reduces torque oscillations, leading to more durable and reliable operation. The smoother curve of Technique 2 indicates that its linear AoA adjustment effectively balances lift and drag, providing steady performance without the large spikes seen in Technique 1. Compared to the fixed-blade configuration (black line), both techniques improve rotor performance by increasing Cp and reducing flow separation and drag. The fixed-blade configuration shows lower Cp values with pronounced dips, highlighting inefficiencies due to suboptimal AoA, leading to reduced torque and energy capture.

The blade pitching models (Techniques 1 and 2) affect instantaneous Cp at different TSRs, as shown in Fig. 11. Both techniques reduce Cp fluctuation amplitude, with Technique 2 offering the most uniform Cp distribution, indicating smoother operation and reduced turbine wear. AoA analysis against blade azimuth position shows that variable pitching controls AoA within an optimal range, minimizing stall risk and enhancing aerodynamic efficiency. Both techniques improve turbine adaptability to varying wind conditions, with Technique 2 demonstrating robustness across diverse wind regimes. These results are attributed to the reduction of cyclic drag forces around the blade and controlled vortex formation downstream, ultimately enhancing turbine performance [46].

Implementing blade pitching techniques not only enhances turbine performance but also improves vibration stability and mechanical durability. Reduced Cp fluctuation amplitude, especially with Technique 2, indicates a balanced operation, potentially reducing vibration and mechanical stress. These improvements could extend VAWT lifespan and decrease maintenance requirements, enhancing reliability and efficiency in the long run.

## 4 Conclusions

This study presents an in-depth analysis of variable blade pitching techniques for a 3-bladed NACA0015 rotor in Vertical Axis Wind Turbines (VAWTs) utilizing the Double Multiple Stream Tube (DMST) theory. By examining two distinct pitching strategies across a comprehensive range of Tip Speed Ratios (TSRs) and wind conditions, this research provides valuable perspectives into the potential of adaptive aerodynamics for enhancing VAWT efficiency. In Technique 1, the angle of attack experienced cyclic variation as a sinusoidal function providing smooth pitching, whereas in Technique 2, the peak value of angle of attack was fixed below the stall condition forming linear function. The primary findings of this study are.

• **Performance Enhancement**: Both techniques significantly improve the performance coefficient (Cp) of VAWTs over fixed-blade configurations, affirming that variable blade pitching can substantially boost energy capture and operational efficiency. Technique 1 achieves higher peak Cp at TSR 5, while Technique 2 provides consistent performance and stability across TSR ranges.
• **Torque Stability and Mechanical Reliability**: Variable blade pitching effectively reduces Cp fluctuation amplitude, contributing to a smoother and more reliable turbine operation. Technique 2, in particular, demonstrates superior torque regulation, reducing mechanical stresses and potential wear, which is critical for long-term structural integrity.
• **Aerodynamic Efficiency and Stall Mitigation**: The dynamic adjustment of the angle of attack (AoA) within an optimal range prevents aerodynamic stall, enhancing lift generation and aerodynamic efficiency. This capability ensures that the blades maintain efficient flow alignment, especially in high-wind upstream conditions.
• **Adaptability Across Wind Regimes**: Both techniques exhibit adaptability to variable wind conditions, enhancing the robustness and versatility of VAWTs in diverse environments. Technique 2's resilience across wind regimes makes it a promising choice for maintaining stable performance under fluctuating conditions.

These findings highlight the potential of variable blade pitching, guided by DMST theory, to transform VAWT technology. Technique 2, with its optimal balance of stability and efficiency, emerges as an especially strong candidate for next-generation VAWT designs, particularly for applications requiring reliable, high-performance wind energy solutions. This work advances the field of renewable energy by offering practical insights into the aerodynamic optimization of VAWTs, positioning variable blade pitching as a viable pathway towards more efficient and sustainable wind energy capture.

Future work could focus on integrating these variable blade pitching techniques within high-fidelity CFD models to optimize VAWT performance in clustered wind farms and study flow physics includes wakes based on fluid solid interaction. By examining aerodynamic interactions among closely spaced turbines, research can enhance positioning and blade control strategies, reduce wake interference, and improve flow recovery, thereby maximizing energy yield and operational stability. Additionally, developing adaptive control algorithms that adjust blade pitch in real time based on wind conditions would increase efficiency and durability by minimizing mechanical stress. Enhancing the DMST algorithm to incorporate wake effects more accurately could also provide deeper understanding for optimizing VAWT arrays.

## CRediT authorship contribution statement

**Antim Gupta:** Writing – original draft, Visualization, Validation, Software, Methodology, Investigation, Formal analysis, Data curation, Conceptualization. **Ussama Ali:** Writing – review & editing, Visualization, Investigation, Formal analysis. **Hamid Ait Abderrahmane:** Writing – review & editing, Writing – original draft, Investigation, Formal analysis. **Isam Janajreh:** Writing – review & editing, Writing – original draft, Supervision, Resources, Project administration, Methodology, Investigation, Funding acquisition, Formal analysis, Data curation, Conceptualization.

## Declaration of competing interest

The authors of this work declare that they have no conflict of interest whatsoever.
